# A validated custom pipeline for three-dimensional kidney stone renderings tocreate an open access repository

**DOI:** 10.1007/s00240-026-02019-9

**Published:** 2026-06-18

**Authors:** Kimberly Cortés Pérez, Joao G. Porto, Luca Civetta, Archan Khandekar, Robert Marcovich, Hemendra N. Shah, Sarvesh Saini, Julio Ojalvo, Ubbo Visser, Jonathan E. Katz

**Affiliations:** 1 Desai Sethi Urology Institute, University of Miami, Miller School of Medicine, Miami, FL USA; 2 Department of Biomechanical Engineering, University of Michigan, Ann Arbor, MI USA; 3 Department of Computer Science, University of Miami, Miller School of Medicine, Miami, FL USA

**Keywords:** Kidney stones, Stone composition, Texture mapping, Digital twin

## Abstract

Three-dimensional (3D) rendering of urologic pathology plays an important role in simulation-based education, surgical training, and computer vision research; however, a standardized, open-access repository of high-fidelity kidney stone models stratified by chemical composition is lacking. We developed and validated a reproducible photogrammetry-based pipeline to generate realistic 3D kidney stone renderings. Chemically characterized human stones composed of calcium oxalate monohydrate (COM) (*n* = 11), uric acid (UA) (*n* = 5), cystine (*n* = 4), magnesium ammonium phosphate hexahydrate/carbonate apatite (MAPH/CA) (*n* = 2), and calcium hydrogen phosphate dihydrate (CHPD) (*n* = 3) were photographed using a custom-built rotating stage and dual fixed 4 K cameras. Rendered models were sent to 25 endourologists using a 5-point Likert-scale survey assessing geometric and surface texture fidelity. Successful 3D renderings were obtained for 8/11 COM stones, 5/5 UA stones, 2/2 MAPH/CA fragments, and 3/3 CHPD fragments, while all cystine stones failed to render. Across stone types, mean fidelity scores were highest for UA and COM stones (mean 3.8–3.9), intermediate for calcium phosphate stones (mean 3.6–3.8), and lowest for struvite stones (mean 3.0–3.3). Geometry scores were higher than texture scores overall, though this difference was not significant. Significant differences in geometric fidelity were observed across stone compositions (χ² = 9.30, *p* = 0.026). Inter-rater reliability was poor for individual evaluators (ICC = 0.10) but moderate for aggregated mean ratings (ICC = 0.67). This validated workflow enables the creation of generally realistic, open-access 3D kidney stone models (*github.com/uro-glidar/3d-rendering-diverse-stones*) for simulation, education, and future machine learning applications in endourology.

## Introduction

Urolithiasis is a common condition, affecting between 1 and 20% of the global population, and is characterized by a high recurrence rate despite appropriate treatment [1]. Management also depends largely on the stone’s composition, which influences both surgical decisions and medical strategies for the prevention of recurrence [2].

Given the high disease burden, mastery of stone management represents an essential component of urologic training. Historically, considerable effort has been devoted to improving trainees’ ability to navigate the upper urinary tract, using physical simulation trainers [3–9]. Relatively, few studies have focused on the feasibility of replicating urinary stones using three-dimensional (3D) modelling [10, 11] and they did not account for variations in stone type.

More recently, 3D simulation models for surgical planning have been utilized across a variety of procedures [12]. However, high-fidelity, open-access simulations that incorporate both the collecting system and realistic kidney stone morphology are lacking. These are necessary building blocks for developing a ureteroscopy with laser lithotripsy (URS) simulation pipeline, which could enable virtual reality training for residents and computer vision training models to facilitate the development of surgical assistance. Therefore, we conducted a study aimed at creating a repository of high-fidelity, 3D-rendered models of commonly occurring kidney stones that can be used for simulation-based training and evaluated the renderings accuracy by surveying endourologists. This resource is expected to enhance the realism of endourologic simulations, improve surgical skill acquisition, and support research and education focused on development of surgical assistance algorithms for URS.

## Methods

Human kidney stones were obtained from the Louis C. Herring Laboratory, where stones from urologic procedures were sent for chemical analysis. Stones were classified as calcium oxalate monohydrate (COM), cystine, magnesium ammonium phosphate hexahydrate/carbonate apatite (MAPH/CA), and calcium hydrogen phosphate dihydrate (CHPD). Except for the fragmented MAPH/CA stone (80% MAPH, 20% CA) and the fragmented CHPD stone (containing 1% hydroxyl apatite), all others were intact and were of a single composition.

**Fig. 1:**
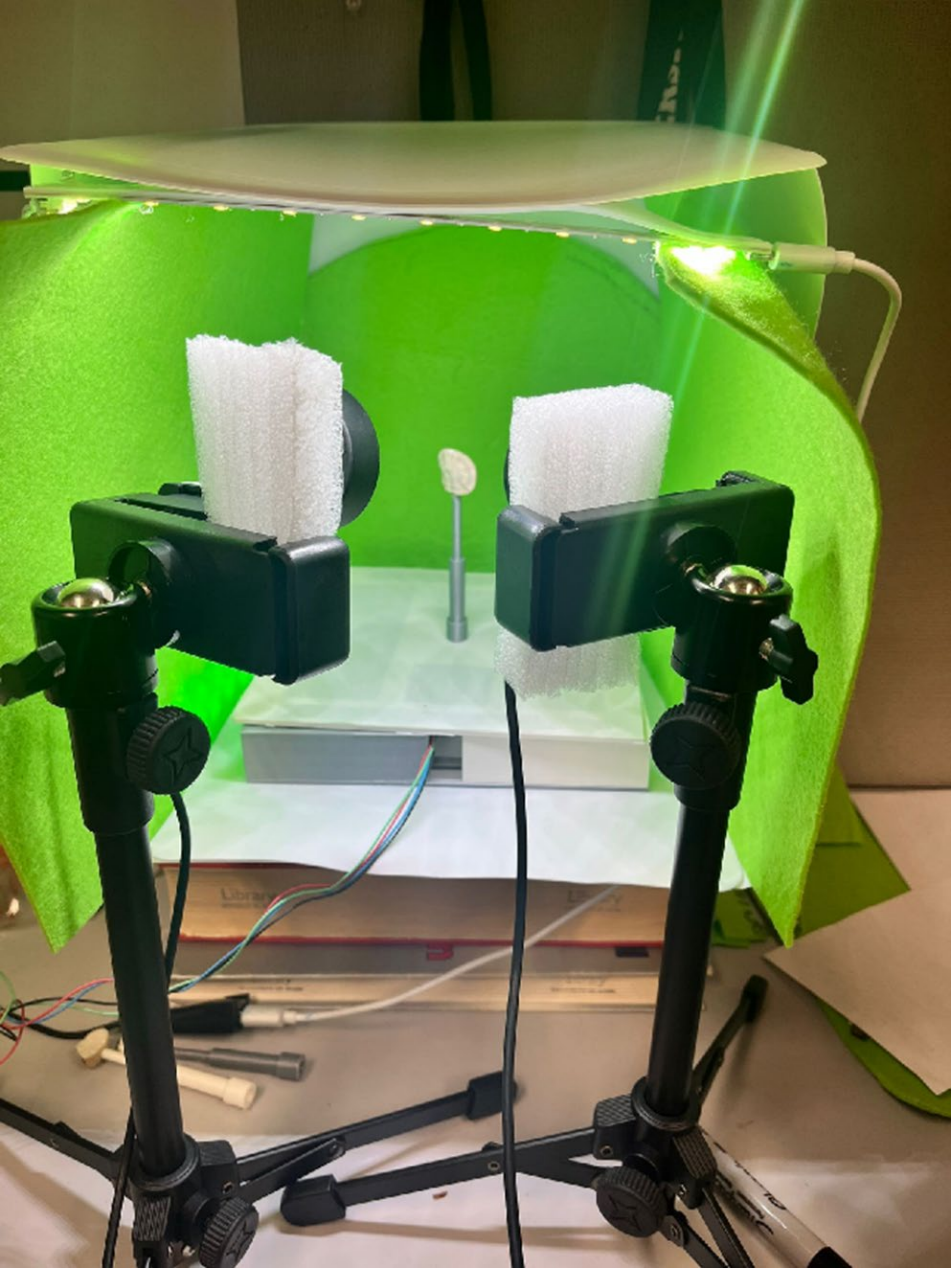
Motorized Stage inside a light box, with two fixed cameras positioned for stereoscopic imaging

Each stone was photographed within a custom-built light box featuring a uniform white, green or black background. A motorized stage was constructed inside the light box, consisting of a NEMA 14 stepper motor (24 V DC, 1.2 A current rating), a 3D-printed polylactic acid (PLA) holder to secure the stone, and two fixed cameras positioned for stereoscopic imaging. The imaging system utilized two EMEET C950 4 K UHD cameras (8-megapixel resolution), placed approximately 7.5 cm apart and 10 cm from the stone (Fig. 1).

Custom control software was developed to synchronize image capture from both cameras. Each stone was photographed 20 times at 18° intervals, producing a full 360° view. Images were captured at 5-second intervals, with the motor manually rotated between shots using Arduino IDE 2.3.6 (ARM 64). The resulting 40 images (20 positions × 2 cameras) were imported into Adobe Substance 3D Sampler version 4.5, utilizing the software’s photogrammetry-based reconstruction pipeline with default parameters. This pipeline automatically aligns overlapping images, estimates camera positions, and generates a textured 3D mesh from the image set without manual parameter adjustment. No preprocessing or image adjustments were performed prior to rendering. Mesh integrity was validated in Blender 4.5.3 using the “Select Non-Manifold “function, which identifies boundary edges, non-manifold edges, and non-manifold vertices. All 19 meshes returned zero non-manifold elements, confirming watertight, manifold geometry suitable for real-time rendering and simulation applications.

During initial optimization, various imaging setups were tested to maximize fidelity and efficiency. A Canon EOS T7i DSLR camera produced clear but inconsistently focused images, while an iPhone 16 Pro Max paired with a 15× macro lens (Ultimate Smartphone Lens Kit) generated high-quality images but required more precise manual alignment and was time-consuming. Ultimately, the dual-webcam setup described above provided the best balance of image quality, reproducibility, and workflow efficiency.

Static images of the kidney stones were sent out to 25 endourologists. Each endourologist was given a Likert rating scale to rate the fidelity of the renderings of each stone type based on geometry and texture with scores from 1 to 5 (1-Strongly Disagree, 2-Disagree, 3-Neutral, 4-Agree, 5-Strongly Agree). For each stone type, we calculated the mean, standard deviation, median and interquartile range. We assessed the data distribution using the Shapiro–Wilk test and because the data were not entirely normally distributed, we used the Mann–Whitney U to compare the difference in accuracy of geometry versus texture overall. We then utilized the Friedman test, to determine whether there was a statistical difference between stone types in accuracy of rendering according to either geometry or texture. Because geometry was statistically significant, we then performed pairwise comparison with the Wilcoxon signed-rank test. Finally, we evaluated the inter-rater agreement using the intraclass correlation coefficient (ICC), with missing data omitted. All statistical analysis was performed with Python 3.13. All 18 stone renderings are available at “*github.com/uro-glidar/3d-rendering-diverse-stones*”.

Each model is provided in Universal Scene Description (USD) format, with four component files per stone: a root assembly file (usd), geometry (geo.usd), material properties (material.usd), and layer composition (layers.usd).

## Results

**Fig. 2:**
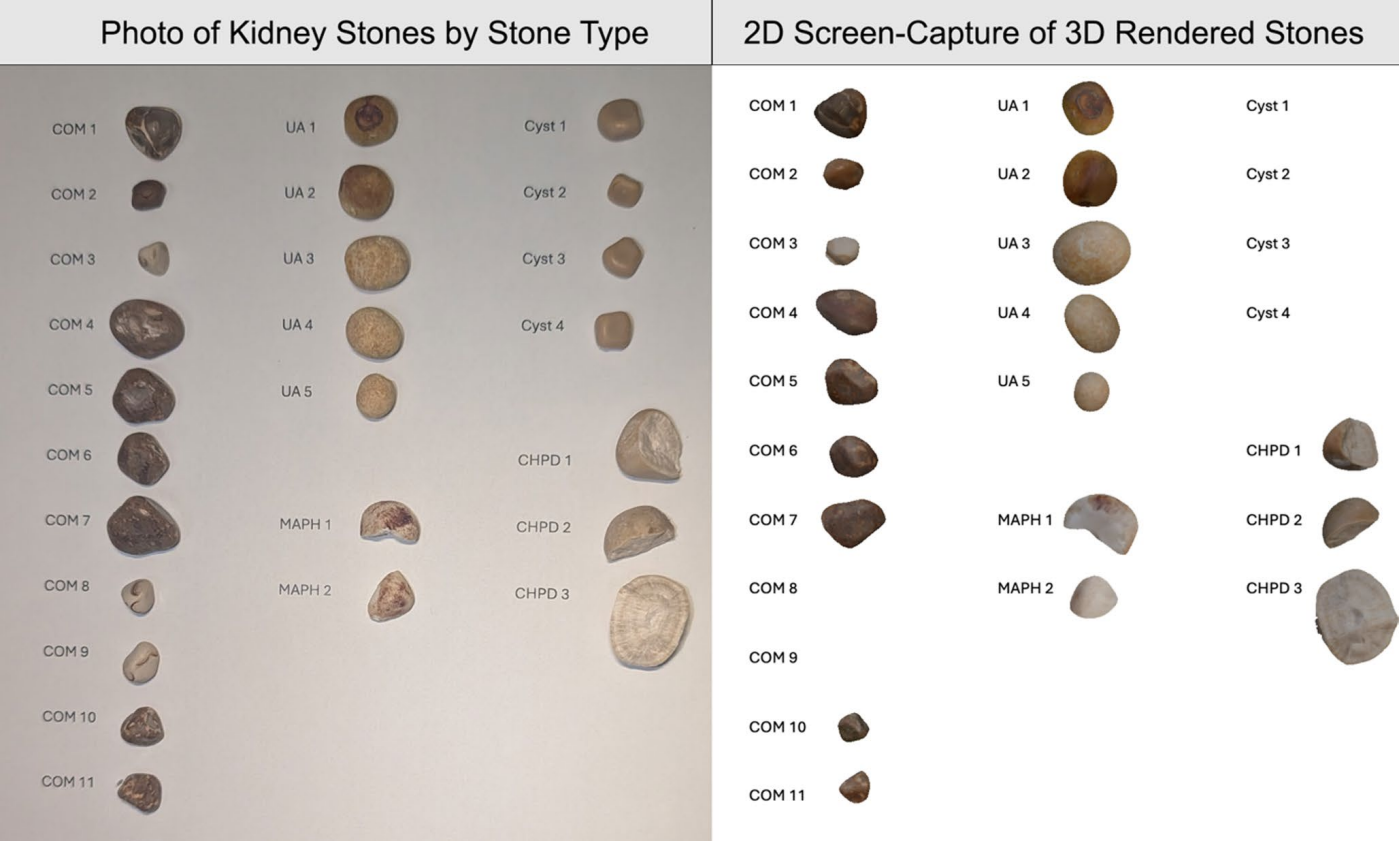
2D image of real stones aligned by stone composition. 2D screen-capture of 3D rendered stones aligned in same position as real stones

3D models were successfully rendered for 5/5 UA stones, 8/11 COM stones, 2/2 MAPH/CA stone fragments, 3/3 fragments of CHPD stones, and 0/4 cystine stones (Fig. 2). Substance 3D Sampler failed to render the four cystine stones and three smaller COM stones despite our attempts to use white, green, and black backgrounds. Eighteen out of the 25 endourologist evaluators assessed the fidelity of the 3D stone renderings using the Likert-scale questionnaire with all but one completing each rating. Descriptive statistics for each stone type and domain are summarized in Table 1.

**Table 1:** Endourologist-rated fidelity of three-dimensional kidney stone renderings by stone composition

COM	Count	Mean (STD)	Median [25–75%]	Shapiro-Wilk
Geometry	18	3.89 (0.90)	3–4.75	*P* = 0.020
Texture	18	3.61 (1.03)	3–4	*P* = 0.33
Uric Acid				
Geometry	18	3.94 (0.87)	3.25–4.75	*P* = 0.016
Texture	18	3.83 (0.85)	3–4	*P* = 0.023
MAPH/CA				
Geometry	18	3.27 (1.01)	2.25–4	*P* = 0.023
Texture	18	3.00 (1.08)	2–3.75	*P* = 0.116
CHPD				
Geometry	18	3.83 (0.78)	3–4	*P* = 0.02
Texture	17	3.58 (0.79)	3–4	*P* = 0.02

COM: Calcium Oxalate Monohydrate; CHPD: Calcium Phosphate; MAPH/CA: Magnesium ammonium phosphate hexahydrate/carbonate apatite; STD: Standard Deviation

Across all stone types, geometry scores (mean: 3.73 ± 0.97) were assessed as more realistic than texture scores (mean: 3.5 ± 1.01), although this difference did not reach statistical significance (Mann–Whitney U = 20.0, *p* = 0.069). Overall, UA stones received the highest mean ratings for both geometry (3.9 ± 0.9) and texture (3.8 ± 0.9). COM (geometry: 3.8 ± 0.9, texture: 3.6 ± 1.0) and CHPD (geometry: 3.6 ± 0.8, texture: 3.6 ± 0.8), followed closely. Conversely, Struvite stones were rated as the least realistic, yielding the lowest mean scores for both geometry (3.3 ± 1.0) and texture (3.0 ± 1.1).

Normality testing using the Shapiro–Wilk test demonstrated non-normal distributions for most geometry and texture variables (*p* < 0.05). When comparing geometry ratings across stone compositions, a Friedman test demonstrated a statistically significant difference among stone types (χ² = 9.30, *p* = 0.026); while no significant difference was noted for texture (χ² = 7.26, *p* = 0.064) (Table 2). Post-hoc pairwise comparisons revealed significantly lower geometry ratings for struvite stones compared with COM (*p* = 0.008) and CHPD (*p* = 0.019). No significant differences were observed between COM, uric acid (UA), and CHPD stones (Table 3).

**Table 2:** Nonparametric comparison of geometry and texture fidelity across stone compositions

Category	U-Statistic	*P*-value
Texture	7.26	0.064
Geometry	9.30	0.026

**Table 3:** Comparisons of geometric fidelity between stone compositions

Comparison Group	U-Statistic	*P*-value
COM vs. UA	27.5	0.59
COM vs. CHPD	20.0	0.74
COM vs. MAPH/CA	0.0	0.0084
UA vs. CHPD	19.5	0.71
UA vs. MAPH/CA	27.0	0.053
CHPD vs. MAPH/CA	11.0	0.019

COM: Calcium Oxalate Monohydrate; CHPD: Calcium Phosphate; MAPH/CA: Magnesium ammonium phosphate hexahydrate/carbonate apatite

Inter-rater reliability was calculated using a two-way random-effects model (ICC2). Although inter-rater reliability for individual evaluators was poor (ICC[2,1] = 0.10, 95% CI [0.02, 0.40], *p* = 0.0006), the reliability of the mean ratings was moderate (ICC[2,k] = 0.67, 95% CI [0.29, 0.92], *p* = 0.0006), indicating acceptable consensus when expert assessments were aggregated.

## Discussion

We developed high-fidelity 3D renderings of kidney stones stratified by chemical composition and described our technique. Using a standardized imaging setup and Adobe Substance 3D Sampler for rendering, we produced realistic models for COM, UA, and CHPD stones, with moderate agreement among endourologists regarding geometric accuracy and acceptable agreement regarding surface texture. Struvite stones had the lowest overall rating, which was statistically significant with regards to geometry. Furthermore, we were unable to render cystine stones, likely due to optical reflectivity rather than pipeline failure, suggesting that further optimization of lighting, surface treatment, or image preprocessing may improve performance. Nonetheless, this work addresses the lack of 3D digital kidney stone assets that are publicly available, since prior kidney stone surgery simulation research focused primarily on collecting system anatomy or isolated printed stone replicas without 3D digital rendering and without systematic validation by stone composition [10, 11, 13].

The observed differences in rendering performance across stone types are biologically and optically plausible. COM and UA stones typically exhibit irregular but matte surfaces, which are well-suited for photogrammetry-based reconstruction. In contrast, cystine stones and small paler COM stones have high surface reflectivity and low textural contrast, which likely interfered with feature detection and depth estimation, resulting in failed renderings [14]. Highly reflective surfaces introduce specular highlights that vary with camera angle and lighting conditions, leading to inconsistent feature matching across frames [15]. Additionally, low-texture or homogeneous surfaces lack sufficient visual features for robust key point extraction, further impairing accurate point cloud reconstruction and mesh generation. Together, these factors can result in incomplete geometry, noisy reconstructions, or total rendering failure [16]. Similarly, the lower geometry scores observed for struvite stones may reflect the perception that they typically have a staghorn appearance, though this was not the case for the specimens we received. Regarding texture, their smoother surfaces and heterogeneous fragmentation patterns may have limited the fidelity of the rendering [17].

Recent advances in kidney stone segmentation using contemporary computer vision pipelines have improved stone segmentation [18] and enabled detection of stone type, size, and number [19]. However, these are extremely labor-intensive techniques which often require hundreds of hours of labeling to prepare data for computer vision models. By developing high fidelity digital renderings of kidney stones, surgical scenes can be created and next generation of computer vision models can be augmented with synthetic data, obviating the need for as much data annotation. This technique has previously been validated in object detection tasks on common household items [20]. Our approach is therefore complementary rather than competitive: photogrammetry-based stone modeling may allow rapid generation of annotated images to further optimize computer vision models in kidney stone surgery. Nonetheless, beyond reducing annotation burden, inclusion of diverse stone compositions, morphologies, and surface properties is essential for computer vision models to generalize across heterogeneous intraoperative scenarios and may further improve automated stone tracking and stone type detection.

Similarly, digital assets of common pathologies may be beneficial for surgical rehearsal and resident training. In the current paradigm, surgical trainees often get their first opportunity to use a ureteroscope in a human patient. However, it has been shown that when trainees get an opportunity to practice their technique ahead of time with either a 3D printed kidney models or porcine models, they demonstrate improvement in ureteroscopic control, which may make surgical training safer [21, 22]. Attempts to develop a virtual reality trainer were first trialed in 2002, but have not yet become widespread in endoscopic training and the fidelity to reality was somewhat lacking [23]. Conversely, in robotic training, the da Vinci Skills Simulator was validated and is more widely utilized as part of training prior to trainees using the actual robot in urologic and colorectal surgery [24–26]. We foresee similar developments in ureteroscopy where in the future residents will be able to first practice with a digital simulation prior to surgery and for this realistic kidney stones will be needed.

This study has several notable strengths. First, it presents a reproducible workflow for developing kidney stone renderings using readily available hardware and software, increasing accessibility for academic centers without specialized imaging infrastructure. Second, the models were validated by practicing endourologists, ensuring clinical relevance rather than purely technical success. Third, stones were classified by true chemical composition, allowing meaningful evaluation across clinically relevant stone types rather than visual approximation alone. Finally, the intent to create an open-access repository enhances transparency, reproducibility, and downstream utility for education, simulation development, and machine learning research. However, the present manuscript also has several limitations. The number of stones available for rendering was limited, particularly for less common compositions, which may restrict generalizability. Additionally, only a single 3D rendering platform was used; alternative photogrammetry or physics-based rendering engines may better handle highly reflective stones.

While individual rater reliability scores were low, in aggregate the simulations were reliably acceptable. The low inter-rater reliability likely reflects variability in subjective perception of stone features, particularly given the use of static images of complex 3D structures. Differences in clinical experience and weighting of visual features may have contributed to this variability. Aggregated expert scores mitigate this effect by reducing individual subjectivity [27]. These findings highlight limitations of perceptual evaluation and support the need for more objective metrics and interactive 3D model assessment in future work. Future modification will be needed for highly reflective pale stones such as some COM and cystine stones. Finally, stones utilized in this study were primarily of pure composition, but in reality, in the US most stones are of mixed composition [28].

## Conclusion

We successfully developed and validated a reproducible pipeline for generating high-fidelity 3D kidney stone models across multiple stone compositions. In the future, we believe these renderings can be used to enhance simulation-based training, stone composition recognition, and surgical education. Rendering failures observed in cystine stones and small pale COM stones were most likely related to unfavourable optical surface properties, such as high reflectivity. Future work can apply this workflow to additional stone types, incorporate advanced rendering platforms, and expand validation metrics to strengthen the development of comprehensive, open-access 3D kidney stone libraries for educational and research applications.

## Data Availability

No datasets were generated or analysed during the current study.
